# Screening and Molecular Mechanisms of Novel ACE-Inhibitory Peptides from *Gracilariopsis lemaneiformis*

**DOI:** 10.3390/ijms232314850

**Published:** 2022-11-27

**Authors:** Yongchang Su, Shicheng Chen, Jiashen Shen, Zhiwei Yi, Shuji Liu, Shuilin Cai, Nan Pan, Kun Qiao, Xiaoting Chen, Bei Chen, Min Xu, Suping Yang, Zhiyu Liu

**Affiliations:** 1 College of Chemical Engineering, Huaqiao University, Xiamen 361021, China; 2 Key Laboratory of Cultivation and High-Value Utilization of Marine Organisms in Fujian Province, National Research and Development Center for Marine Fish Processing (Xiamen), Fisheries Research Institute of Fujian, Xiamen 361013, China; 3 Department of Clinical and Diagnostic Sciences, School of Health Sciences, Oakland University, Rochester, MI 48309, USA; 4 College of Food Science, Fujian Agriculture and Forestry University, Fuzhou 350002, China; 5 Technology Innovation Center for Exploitation of Marine Biological Resources, Third Institute of Oceanography, Ministry of Natural Resources, Xiamen 361005, China

**Keywords:** *Gracilaria lemaneiformis*, ACE-inhibitory activity, virtual screening, mechanism, antihypertensive activity

## Abstract

Candidate peptides with novel angiotensin-I-converting enzyme (ACE) inhibitor activity were obtained from hydrolysates of *Gracilariopsis lemaneiformis* by virtual screening method. Our results showed that *G. lemaneiformis* peptides (GLP) could significantly lower blood pressure in spontaneously hypertensive rats (SHR). At least 101 peptide sequences of GLP were identified by LC-MS/MS analysis and subjected to virtual screening. A total of 20 peptides with the highest docking score were selected and chemically synthesized in order to verify their ACE-inhibitory activities. Among them, SFYYGK, RLVPVPY, and YIGNNPAKG showed good effects with IC_50_ values of 6.45 ± 0.22, 9.18 ± 0.42, and 11.23 ± 0.23 µmoL/L, respectively. Molecular docking studies revealed that three peptides interacted with the active center of ACE by hydrogen bonding, hydrophobic interactions, and electrostatic forces. These peptides could form stable complexes with ACE. Furthermore, SFYYGK, RLVPVPY, and YIGNNPAKG significantly reduced systolic blood pressure (SBP) in SHR. YIGNNPAKG exhibited the highest antihypertensive effect, with the largest decrease in SBP (approximately 23 mmHg). In conclusion, SFYYGK, RLVPVPY, and YIGNNPAKG can function as potent therapeutic candidates for hypertension treatment.

## 1. Introduction

Hypertension is associated with many serious diseases, including cardiovascular disease, heart attack, atherosclerosis, and kidney disease [1]. As one of the most leading causes of death and morbidity, it sickens up to 30% of the adult population [2]. ACE plays an essential role in the maintenance of blood pressure [3]. The enzyme converts angiotensin I into angiotensin II (a vasoconstrictor) in the renin–angiotensin system (RAS), and inactivates bradykinin and kallidin (vasodilators) in the kallikrein–kinin system (KKS) [4,5]. Therefore, inhibitors of ACE activity can be used to manage hypertension. A variety of synthetic ACE inhibitors, including captopril, enalapril, and lisinopril fosinopril have been extensively employed in attempted hypertension treatments [6]. Unfortunately, synthetic ACE inhibitors often have adverse effects on patients, including hypotension, dry cough, dizziness, fatigue, and elevated blood potassium levels [7,8]. Therefore, studies on alternative sources of synthetic ACE inhibitors may need to be warranted [9].

ACE-inhibitory peptides demonstrated great therapeutic potential to prevent hypertension. Many potent ACE-inhibitory peptides have been acquired from natural foods, including meat [10], milk proteins [11], fish [12], plants [13], and macroalgae [14]. Marine algae proteins are considered to be a good source of producing bioactive peptides. For example, two new ACE-inhibitory peptides purified from *Bangia fuscopurpurea* demonstrated good resistance to enzymatic degradation [15]. Furthermore, Sun et al. isolated ACE-inhibitory peptides from the marine macroalga *Ulva intestinalis* by ultra-filtration, gel chromatography, and HPLC methods [16].

*G. lemaneiformis,* belongs to the family *Gracilariaceae*, which is widely cultivated in Asian countries, including China, Japan, and Korea [17,18]. It is widely used for food products, feedstock for abalone, and raw industrial material (i.e., agar) [19]. The current research on *G. lemaneiformis* mainly focuses on the extraction of high-value products (polysaccharides) [20] and nutritional compositions [21] as well as other pharmaceutical products. Particularly, *G. lemaneiformis* peptides possess ACE-inhibitory activity as well as an antioxidant capacity [21,22].

In this study, we extracted GLPs using enzymatic hydrolysis and ultrafiltration methods. Several potential ACE-inhibitory peptides of GLP were identified and screened by LC-MS/MS and virtual screening. A molecular simulation study was carried out to elucidate their mechanism against ACE. We demonstrated that they may significantly reduce systolic blood pressure (SBP) in SHR.

## 2. Results and Discussion

### 2.1. ACE-Inhibitory Activity and Antihypertensive Effect of GLP

**Figure 1:**
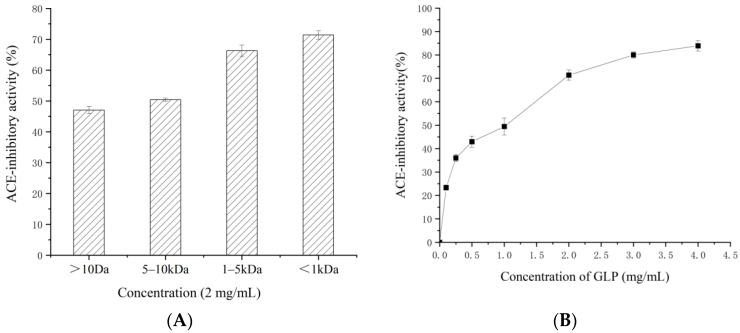
ACE-inhibitory activity of different MW fractions (**A**) and <1 kDa fractions (**B**).

GLPs were separated using an ultrafiltration method by running *G. lemaneiformis* hydrolysates through 1, 5, and 10 kDa MWCO membranes. Four fractions (molecular weights ranges: <1 kDa, 1–5 kDa, 5–10 kDa, and >10 kDa) inhibited ACE activities by 71.37 ± 0.22%, 66.3 ± 1.82%, 50.4 ± 0.50%, and 47.07 ± 1.18%, respectively (Figure 1A). The fraction with molecular weight less than 1 kDa exhibited the highest ACE-inhibitory activity. Its lowest half-maximal inhibitory concentration (IC_50_) was estimated to be 0.62 mg/mL (Figure 1B).

It is well documented the bioactivity of GLP is affected by its molecular size [23,24]. Low-MW peptides demonstrated the potential to adopt to specific three-dimensional structures. Thus, it allows them to bind to the amino acid residues on the ACE active site, which leads to enzyme inactivation [25,26]. Our results showed that low-MW peptides in *G. lemaneiformis* extracts inhibited more ACE activity than did high-MW peptides, which was consistent with previous observation [27]. Therefore, the GLP fraction with a molecular weight less than 1 kDa was chosen for further analysis.

### 2.2. Antihypertensive Activity of the GLP in SHR

**Figure 2:**
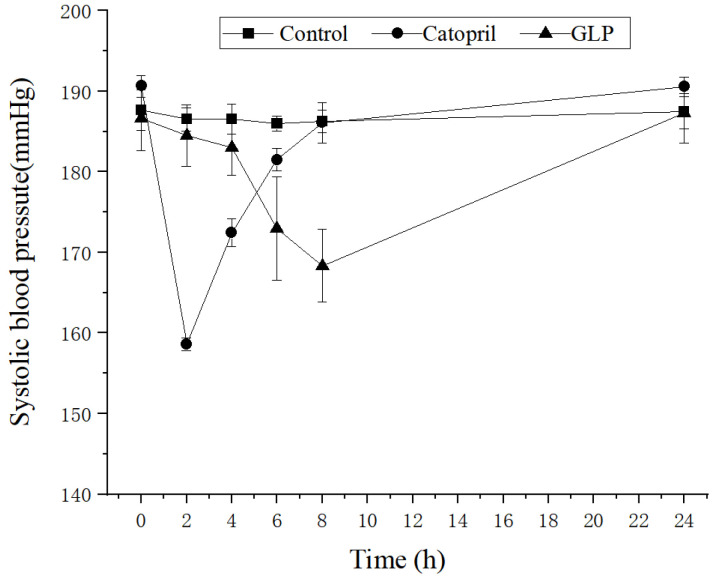
Systolic blood pressure changes in spontaneously hypertensive rats after the oral administration of *G. lemaneiformis* peptide.

The antihypertensive efficacy of GLP was evaluated in spontaneously hypertensive rats (SHR). Systolic blood pressure (SBP) in the control group fluctuated between 185 and 187 mmHg, showing that the tested animals had hypertension. The blood pressure reduction was first observed (~10 mmHg lower than the control) in the GLP-treated SHRs after 4 h (*p* < 0.01). The GLP demonstrated a maximum antihypertensive effect (~20 mmHg lower than the control) after 8 h. However, SBP increased to 191 mmHg after 24 h. Our results further demonstrated that GLP treatment allowed for the maintenance of lower SBP levels in a relatively longer period if compared to the group treated by captopril. It should be noted that captopril reached its maximum effect faster than GLP (Figure 2).

The delivery of antihypertensive peptides by the oral route presented many challenges. For example, the gastrointestinal proteases, the low pH environment in the stomach, and the efficiency of intestinal absorption often affect the performance of antihypertensive peptides [28]. Oral drugs are generally not bioavailable or bioactive due to these factors. Previous research shows that ACE-inhibitory peptides isolated from food protein hydrolysates have demonstrated excellent ACE-inhibitory activity as well as great bioavailability [24].

### 2.3. Identification and Virtual Screening of the ACE-Inhibitory Peptide from GLP

**Figure 3:**
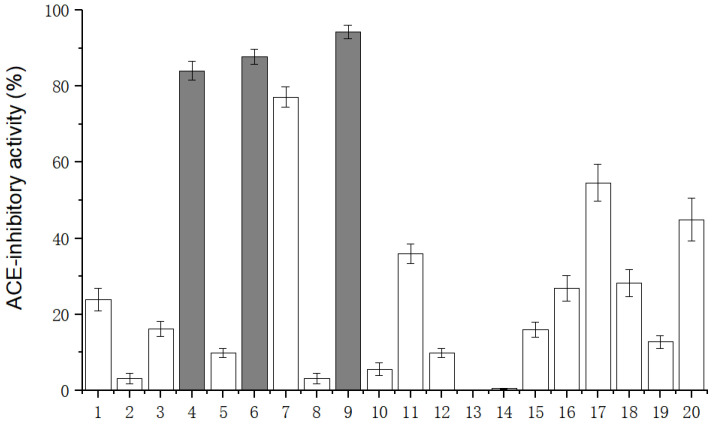
The ACE-inhibitory activity of synthetic peptides. The numbers represent the candidate peptides whose sequences are shown in Table 1.

**Figure 4:**
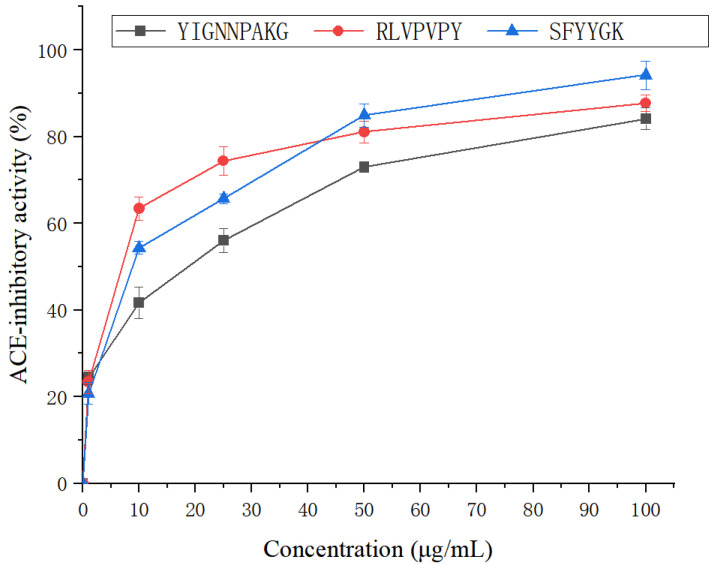
The ACE-inhibitory activity of the three candidate peptides (SFYYGK, RLVPVPY, and YIGNNPAKG).

A total of 101 peptides were proceeded to virtual screening (VS). Among them, 20 peptides (Table 1) with the highest grid score (ranging from −120.504 to −187.632.) were chosen for the determination of ACE-inhibitory activity. The peptides were chemically synthesized, and their ACE-inhibitory activities were investigated (Figure 3). SFYYGK exhibited the highest ACE-inhibitory activity (94.21%), followed by RLVPVPY (87.74%) and YIGNNPAKG (84.09%). Their IC_50_ values were 6.45 ± 0.22, 9.18 ± 0.42, and 11.23 ± 0.23 µmoL/L, respectively (Figure 4).

VS is an increasingly important computational tool for drug discovery. It is faster, more cost-effective and less resource-intensive than traditional screening methods [29]. Recently, VS techniques have been widely used for the discovery of ACE-inhibitory peptides. For example, Lin et al. predicted ACE-inhibitory peptides by establishing four QSAR models [30]. Yu et al. evaluated the ACE-inhibitory activity in bio-peptides derived from myosin using several in-silico methods, and identified a novel peptide [31]. However, it is noted that VS presents some disadvantages including a relatively low accuracy of predictions and a rapid accumulation of errors. Laboratory experiments are warranted to evaluate the virtual screening results [32].

### 2.4. Molecular Mechanisms of the Interactions between Peptides and ACE

**Figure 5:**
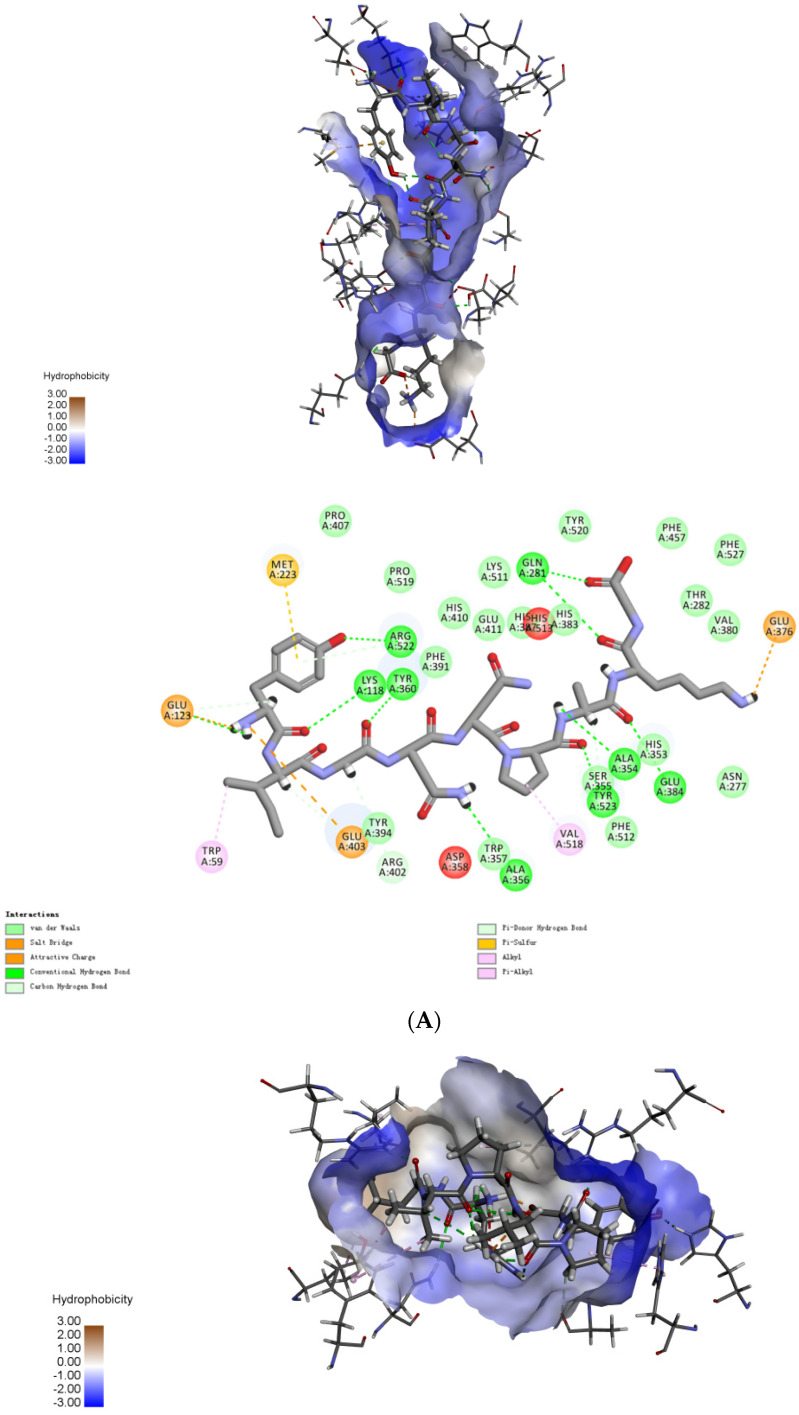

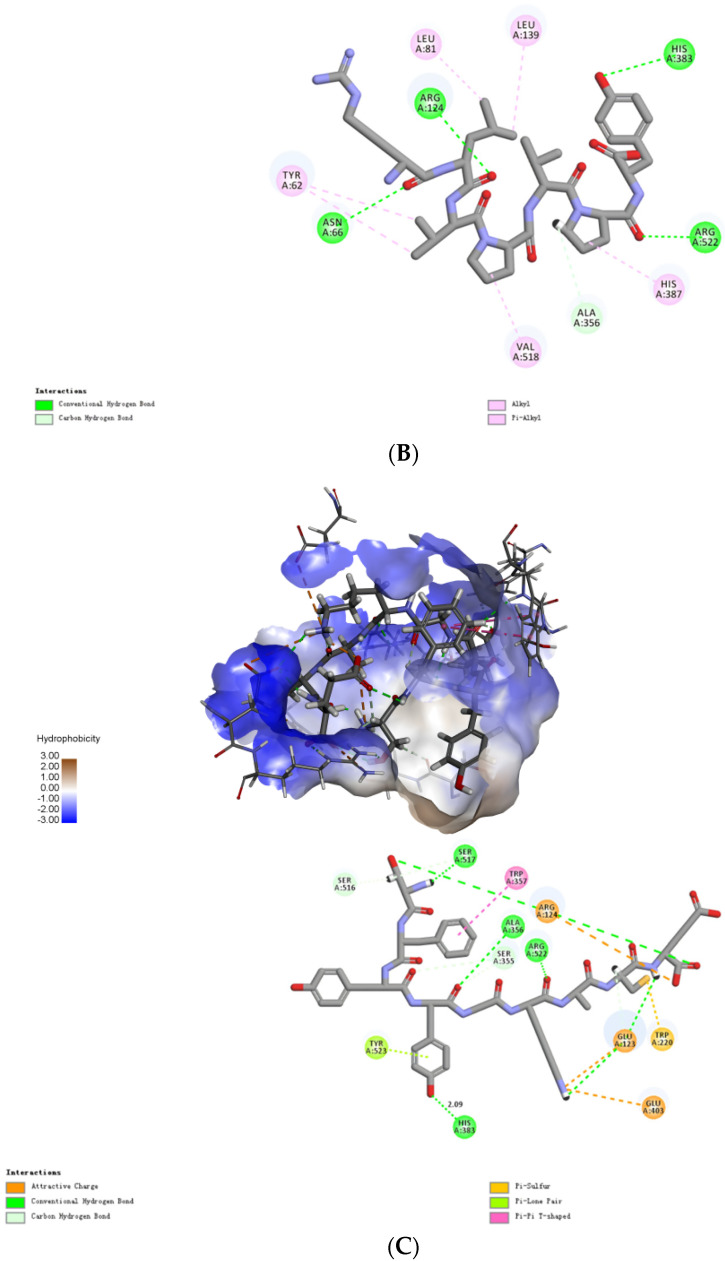

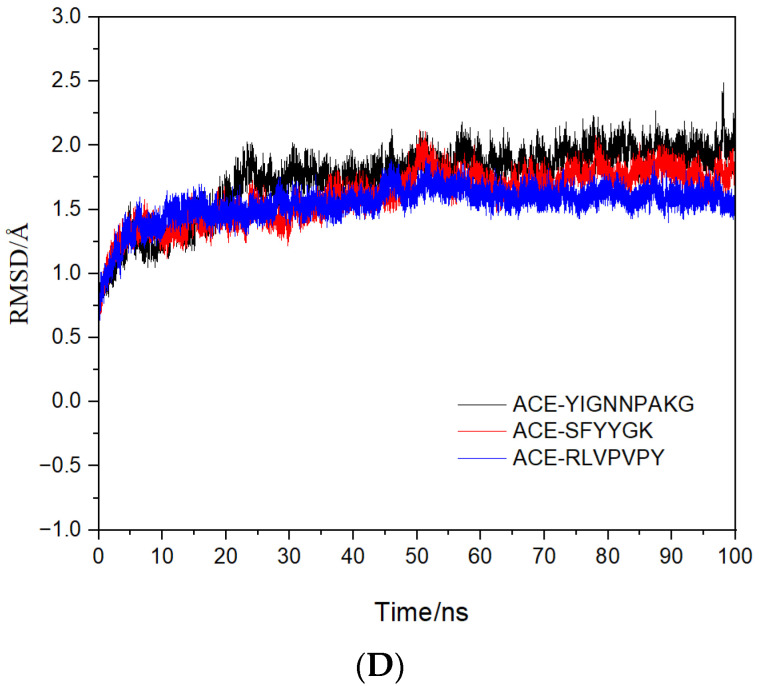
Molecular simulation of the interactions between peptides and ACE: Molecular docking of (**A**) SFYYGK, (**B**) RLVPVPY and (**C**) YIGNNPAKG; (**D**) the changes in RMSD between ACE−peptide complexes.

There were at least three main active site pockets in ACE: Gln281, His353, Lys511, and His513 in S1 pocket; Ala354, Glu384, and Tyr523 in S2 pocket; and Glu162 in S1 pocket. These site pockets are critical for the binding of ACE-inhibitory peptides [33]. Our molecular docking studies showed that SFYYGK, RLVPVPY and YIGNNPAKG bind to the active site pocket of ACE via multiple forces (conventional hydrogen bonding, carbon-hydrogen bonding, Van der waals, salt bridge formation, charge attraction, Pi-Donor hydrogen bonding, Pi-sulfur bonding, Alkyl bonding, and Pi-Alkyl bonding) (Figure 5).

It has been reported that the hydrogen bond interactions play a crucial role in the formation of the stable structure of the ACE-peptide complex [34]. SFYYGK interacted with residues Gln281, Glu384, Ala354, Tyr523, Ala356, Lys118, Tyr360, and Arg522 via nine hydrogen bonds (Figure 5A). Four hydrogen bonds were formed between RLVPVPY and ACE (Asn66, Arg124, His383, and Arg522). YIGNNPAKG formed four hydrogen bonds with residues of ACE (Ala356, His383, Arg522, and Ser517). The above results were consistent with the previous ACE-inhibitory activity assays for SFYYGK, RLVPVPY and YIGNNPAKG.

MD simulations were used to determine their stabilities. Root mean square deviation (RMSD) of the ACE–peptide complexes initially exhibited a large transition. It started to level off and eventually floated below 2 Å (Figure 5D). The binding energies of SFYYGK, RLVPVPY and YIGNNPAKG were −148.371, −96.815, −172.778 kcal/mol, respectively. The MD simulations revealed that the binding processes of SFYYGK, RLVPVPY and YIGNNPAKG reached equilibrium quickly and presented stable binding mode.

### 2.5. Antihypertensive Activity of SFYYGK, RLVPVPY and YIGNNPAKG

**Figure 6:**
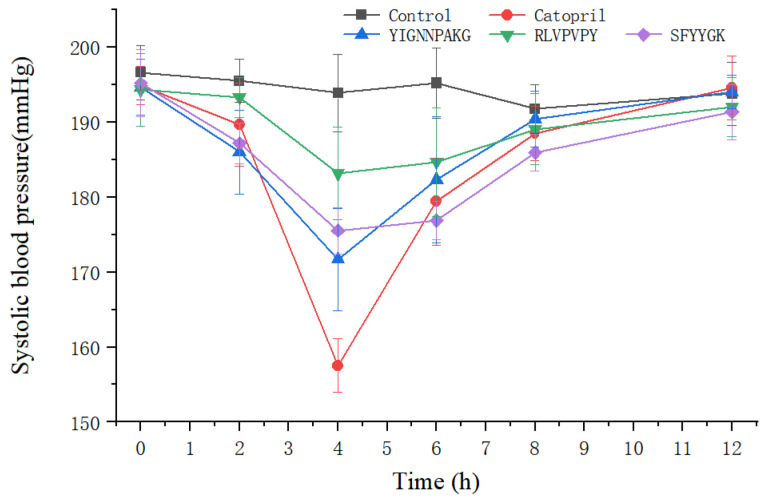
SBP changes in SHRs after the intravenous administration of SFYYGK, RLVPVPY and YIGNNPAKG.

The antihypertensive effects of candidate peptides (SFYYGK, RLVPVPY and YIGNNPAKG) were evaluated in SHR after intravenous administration. As shown in Figure 6, all of YIGNNPAKG, SFYYGK, and RLVPVPY exhibited good blood pressure lowering effects. YIGNNPAKG significantly reduced the SBP between 2 to 8 h (*p* < 0.05), reaching its lowest SBP of 171 mmHg at 4 h. The SBP then recovered to 190 mmHg after 8 h. The SFYYGK group decreased SBP to the lowest level after 4 h by approximately 20 mmHg of SBP from 195 mmHg to 175 mmHg. RLVPVPY showed the least effective antihypertensive effect with the greatest decline (~11 mmHg). All three treatments showed good anti-hypertensive effects. However, YIGNNPAKG and SFYYGK exhibited a higher ability in lowering blood pressure in hypertensive rats than RLVPVPY. Thus, YIGNNPAKG, SFYYGK, and RLVPVPY can serve as potent candidates for the treatment of hypertension. The novel peptide drug candidates could be a lead compound for further truncation, modification, and optimization in order to improve biological activity and pharmacokinetic properties [35].

## 3. Materials and Methods

### 3.1. Materials and Chemicals

*G. lemaneiformis* was supplied by Fujian Sea Biological Polytron Technologies Inc (Zhangzhou, China). Alcalase (EC 3.4.21.62) was provided by China Pangbo Biological Engineering Co., Ltd. (Nanning, China). Trifluoroacetic acid (TFA), hippuryl-l-histidyl-l-leucine (HHL), ACE from rabbit lung (EC3.4.15.1), captopril (>99% purity) was purchased from Sigma-Aldrich (St. Louis, MO, USA). Acetonitrile (ACN, HPLC grade) was purchased from Merck (Darmstadt, Germany). All other reagents and chemicals were analytical grade.

### 3.2. Preparation of G. lemaneiformis Peptides (GLP)

*G. lemaneiformis* was enzymatically hydrolyzed with alcalase at 55 °C for 3 h at a solid-liquid ratio of 1:5 and an enzyme concentration of 1000 U/g (enzyme/substrate). After hydrolysis, centrifugation was carried out at 7104 g at 4 °C for 30 min. The supernatant was subjected to the ultrafiltration process (STAR Biotechnology, Xiamen, China) using ultrafiltration membranes (MWCO:1, 5, and 10 kDa). Fractions of different MW (<1 kDa, 1–5 kDa, 5–10 kDa, and >10 kDa) were collected and lyophilized. The fractions with higher ACE activity were selected for the identification of peptide sequences.

### 3.3. Assay of ACE-Inhibitory Activity

The ACE-inhibitory activity was determined according to the method of Ma et al., with some modification [36]. Briefly, 50 µL of sample in 0.1 mol/L borate buffer (pH 8.3, containing 0.3 M NaCl) was mixed with 150 µL of 5 mM hippuryl-l-histidyl-l-leucine (HHL) and incubated at 37 °C for 10 min. Then, the reaction was initiated by adding 50 µL of ACE solution (50 mU/mL) and incubating at 37 °C for 45 min. The reaction was then stopped by the addition of 250 µL of 1 M HCl. The formation of hippuric acid (HA) was measured by RP-HPLC (Waters, Milford, MA, USA) at 220 nm. The IC_50_ value represents the 50% inhibition ratio of ACE activity under the assay conditions.

### 3.4. Antihypertensive Effect of GLP

SHRs (male, 10 weeks, 220 ± 20 g body weight) were supplied by Vital River Laboratory Animal Technology Co., Ltd. (Beijing, China). SHRs with tail SBP over 180 mmHg were randomly divided into groups of 10 rats and housed at 25 °C under a 12 h light/dark cycle. Three doses of GLP (1200, 1500, 1800 mg/kg of body weight, BW) was orally administered to SHRs. The blank group (saline) and Captopril (30 mg/kg·bw) group were used as negative and positive control, respectively. BP-98A blood pressure monitor (Softron Biotechnology, Beijing, China) was used to determine blood pressure by the tail-cuff method. The SBP of the rats was measured at five points: 2 h, 4 h, 6 h, 8 h, and 24 h post-administration. Animals were treated in accordance with the guidelines of the National Institutes of Health and Use of Laboratory Animals.

### 3.5. Identification and Virtual Screening of the ACE-Inhibitory Peptide

For peptide sequence identification, GLP was analyzed using Q-Exactive mass spectrometer (Thermo Fisher, Waltham, MA, USA). Device parameters referenced the method of Tu et al. [37]. After desalination, the sample was loaded onto an Acclaim PepMap RPLC C_18_ (75 m i.d. × 150 mm). Mobile phase A contained 2% ACN (with 0.1% formic acid, *v*/*v*), and mobile phase B consisted of 80% ACN (with 0.1% formic acid). Gradient elution was performed with a gradient of 6–95% B; the flow rate was 300 nL·min^−1^. The MS data were then searched for the corresponding database using PEAKS Studio (Bioinformatics Solutions Inc., Waterloo, Canada). From the hundreds of generated peptides, only peptides with a confidence score (−10lgP) > 20 were chosen to proceed to the following step. Virtual screening (VS) was performed using the UCSF Dock 6.9 (University of California, San Francisco, CA, USA) in the Yinfo Cloud Platform (https://cloud.yinfotek.com/, (accessed on 2 August 2022)). The mdb file of human ACE (PDB code: 1O8A) was downloaded from the RCSB Protein Data Bank (PDB, https://www.rcsb.org/structure/1O8A, (accessed on 2 August 2022)). The steps were performed according to instructions provided by the DOCK 6.9 software package (www.dock.compbio.ecsf.edu, (accessed on 2 August 2022)). A semi-flexible docking procedure was executed with DOCK 6.9, and the output poses were evaluated using grid scoring [38,39]. Those peptide sequences with higher grid scores were selected and used for chemical synthesis.

### 3.6. Peptide Synthesis and Individual Bioactivity Assays

The screened and predicted potential ACE-inhibitory peptides were chemically synthesized at Genscript biotech corporation (Nanjing, China) using the Fmoc solid-phase method. The sequence and purity (>98%) of synthesized peptides were verified by HPLC and mass spectrometry. Synthetic peptides were dissolved at 1 mg/mL for validation of their ACE-inhibitory activity.

### 3.7. Molecular Mechanisms

The molecular interactions of peptides with ACE were studied by molecular docking and molecular dynamic simulations. Molecular docking was carried out using the Discovery Studio 2019 (NeoTrident Technology, Beijing, China) to investigate the binding mode between peptides and ACE. For ACE docking, the 3D structure of human ACE (PDB ID: 1O8A) was retrieved from the RCSB Protein Data Bank (PDB, https://www.rcsb.org/structure/1O8A, (accessed on 15 September 2022)). Hydrogen atoms were added to the protein structure and the water molecules were removed [40]. The CHARMM force field was used for energy minimization before molecular docking. The best-docked pose was selected to analyze the molecular interactions between ACE and peptides at the ACE active sites [41].

The molecular dynamics (MD) simulations were carried out on the Yinfo Cloud Computing Platform (CCP) using AmberTools 20 (https://cloud.yinfotek.com/, (accessed on 15 September 2022)). ACE and peptides were modeled using AMBER ff19SB and GAFF force fields, respectively [42]. To study the energy contributions of ACE–peptide interaction, 100 ns MD simulation runs were carried out. A total of 200 snapshots were extracted from the MD trajectory and applied for MM/PBSA binding free energy ($∆G_{\mathrm{bind}}$) calculation using the following equation:

$$∆G_{\mathrm{bind}}=∆G_{\mathrm{complex}}-\left(∆G_{\mathrm{receptor}}-∆G_{\mathrm{ligand}}\right)$$

### 3.8. Antihypertensive Effect In Vivo

SHRs were grouped and housed in the same conditions as Method 3.4. FYYGK, RLVPVPY, and YIGNNPAKG was injected via tail vein at a dose of 10 mg/kg body weight. Captopril (5 mg/kg) and saline at the same dose served as positive control and blank control group. Systolic blood pressure was measured before and after intravenous administration by the tail-cuff method. Ethics statement animal studies were approved by the Ethics Committee of Guangdong medical laboratory animal center (no. 20211001).

### 3.9. Statistical Analysis

Data analysis was carried out with SPSS V19.0 (SAS Institute Inc., Cary, NC, USA). ANOVA was used to analyze the experimental data and expressed as means ± standard deviations (SD). Graphs were produced using Origin 2022 (Origin Lab, Northampton, MA, USA).

## 4. Conclusions

In this study, at least 101 peptide sequences of GLP were identified by LC-MS/MS and PEAKS Studio. Following the virtual screening studies carried out, 20 peptides were selected and subjected to an ACE-inhibitory activity assay. Three potential ACE-inhibitory peptides (SFYYGK, RLVPVPY, and YIGNNPAKG) possessed the highest ACE-inhibitory activity with IC_50_ values of 6.45 ± 0.22, 9.18 ± 0.42, and 11.23 ± 0.23 µmoL/L, respectively. The results of molecular simulations suggested that the three peptides could bind ACE and form a stable ACE–peptide complex. Further, animal experiments demonstrated that SFYYGK, RLVPVP, and YIGNNPAKG could significantly decrease the SBP of SHR, which indicates promising anti-hypertensive effects of the peptides.

## Figures and Tables

**Table 1:** The 20 peptides with the highest grid score by virtual screening.

No	Peptide Sequence	Length	Mass (Da)	−10lgP	Grid Score (kJ/mol.)
1	YDYIGNNPAKG	11	1210.562	38.32	−187.632
2	YIGNNPAKGGLF	12	1249.646	41.35	−186.059
3	RELIIGDR	8	970.556	30.57	−162.231
4	YIGNNPAKG	9	932.472	25.92	−160.014
5	SYPGIKF	7	810.428	27.52	−154.084
6	RLVPVPY	7	842.501	24.24	−149.115
7	NYPAWGY	7	869.371	27.91	−147.764
8	TGDSNNN	7	720.268	28.5	−144.587
9	SFYYGK	6	763.354	25.63	−141.493
10	APTHPIRL	8	903.529	30.66	−141.45
11	FFFK	4	587.311	25.43	−139.879
12	SIYVSLPF	8	924.496	24.61	−139.56
13	AYPGDVF	7	767.349	25.54	−137.737
14	FQEPNPI	7	843.413	30.31	−132.76
15	WEK	3	461.2274	30.09	−126.44
16	WWK	3	518.2642	39.07	−125.302
17	KWW	3	518.2642	36.8	−123.22
18	LLVR	4	499.3482	24.75	−123.037
19	WKPW	4	615.3169	25.49	−122.007
20	KIYP	4	519.3057	23.97	−121.024

## Data Availability

Not applicable.
